# Low b-values in apparent diffusion coefficient calculations overestimate diffusion in rectal cancer

**DOI:** 10.2340/1651-226X.2025.44028

**Published:** 2025-10-19

**Authors:** Johanna A. Hundvin, Marius Bornstein, Anne Negård, Stein H. Holmedal, Sebastian Meltzer, Anne H. Ree, Sara Pilskog, Kathrine R. Redalen

**Affiliations:** a Cancer Clinic, Haukeland University Hospital, Bergen, Norway; b Institute of Physics and Technology, University of Bergen, Bergen, Norway; c Department of Oncology, Akershus University Hospital, Lørenskog, Norway; d Department of Radiology, Akershus University Hospital, Lørenskog, Norway; e Institute of Clinical Medicine, University of Oslo, Oslo, Norway; f Department of Physics, Norwegian University of Science and Technology, Trondheim, Norway

**Keywords:** Magnetic resonance imaging, consensus, prospective study, tumour, perfusion, uncertainty

## Abstract

**Background and purpose:**

The apparent diffusion coefficient (ADC), derived from diffusion-weighted MRI (DWI), is commonly calculated using a monoexponential model. However, there is no consensus on optimal *b*-value selection for ADC quantification in rectal cancer. This prospective observational study evaluated how varying *b*-value combinations influence ADC values.

**Patient/material and methods:**

DWI with seven *b*-values (*b* = 0, 25, 50, 100, 500, 1,000, and 1,300 s/mm^2^) was acquired from 23 rectal cancer patients in the OxyTarget study (NCT01816607) using a 1.5T Philips Achieva scanner. Two radiologists independently delineated whole-tumour volumes of interest. ADC values were calculated using 18 different *b*-value combinations and compared with a biexponential reference.

**Results:**

Tumour ADCs varied significantly across *b*-value combinations. Excluding low *b*-values (*b* ≤ 100 s/mm²) led to reduced ADCs. Although *b* = 0 s/mm² is commonly included in ADC calculations, this study demonstrates that its inclusion leads to substantial overestimation. The use of two or three *b*-values from *b* = 500, 1,000, and 1,300 s/mm² yielded the smallest deviations from the biexponential reference.

**Interpretation:**

In rectal cancer, tumour ADC calculated using the monoexponential model is strongly influenced by the choice of *b*-values. By eliminating the contribution from perfusion (*b* ≤ 100 s/mm^2^) the uncertainty in the calculations is significantly reduced. Our findings support the use of *b*-values exceeding 100 s/mm², ideally in combination with a high *b*-value of at least 1,000 s/mm², when assessing diffusion using the monoexponential model. Consistent *b*-value combinations across studies are recommended for reliable quantitative comparisons of ADC values.

## Introduction

Diffusion-weighted MRI (DWI) is a non-invasive functional imaging technique that visualises the random (Brownian) motion of water molecules within tissues [1]. This molecular mobility is impeded by microscopic cellular structures – such as membranes, macromolecules, fibres, blood vessels, and organelles – resulting in reduced water diffusion. Consequently, various characteristics of the tumour microenvironment, including cell density, tissue hypoxia, and vascular architecture and function, influence the DWI signal. In rectal cancer, DWI has emerged as a valuable non-invasive imaging modality with multiple clinical applications. The European Society of Gastrointestinal and Abdominal Radiology (ESGAR) recommends that DWI is routinely performed, particularly for restaging to assess response to preoperative chemoradiotherapy (CRT) [2].

Diffusion is quantified at the voxel level by calculating the apparent diffusion coefficient (ADC) [3], requiring at least two image series with different diffusion-weighting, known as *b*-values. Although not yet widely adopted in routine clinical practice, research has demonstrated its potential in evaluating tumour aggressiveness [4, 5], detection of lymph nodes [6], and prediction of response to preoperative CRT [7]. The reduction in signal intensity (SI) with increasing *b*-value is usually modelled using a monoexponential function, which also applies in the context of rectal cancer. However, reported ADC values in rectal cancer have varied substantially [8], likely due to the lack of consensus on optimal *b*-value selection for quantitative DWI, which limits the comparability of ADC values across patient cohorts. At present, ESGAR does not recommend the use of ADC as a quantitative measure [2].

Although the monoexponential model offers the advantage of simplicity, it does not account for the contribution of perfusion effects. Since signal attenuation due to blood perfusion is particularly pronounced at low *b*-values (< 100–150 s/mm²), it has been proposed to exclude these from the monoexponential model to improve the accuracy of ADC estimation [9]. Nevertheless, many studies continue to include unweighted images (*b* = 0 s/mm²) in ADC calculations [10–15]. As scan time increases with the number of *b*-values, particularly at higher *b*-values, this approach likely reflects an effort to minimise scan duration and reduce motion artefacts caused by patient movement, in addition to adherence to established protocol settings.

The use of a biexponential model, which separates perfusion-related fast diffusion from true molecular diffusion, may provide a more accurate representation of tissue diffusivity when low *b*-values are included [16]. Although not yet established as a clinical gold standard, partly due to its complexity and the requirement for multiple *b*-value acquisitions, studies suggest that the biexponential model may offer improved reliability in quantitative diffusion analysis [3, 17, 18].

Given the considerable inconsistency in *b*-value selection and the notable impact of perfusion-related signal contamination at low *b*-values, the aim of this study was to investigate how different *b*-value combinations within the monoexponential model affect the resulting ADC, with the biexponential model serving as a comparative benchmark.

## Patients/material and methods

### Patients and eligibility

The investigation was performed as part of the prospective biomarker study *OxyTarget – Functional MRI of Hypoxia-Mediated Rectal Cancer Aggressiveness (NCT01816607)*, approved by the Institutional Review Board and the Regional Committee for Medical and Health Research Ethics Southeast. For all patients, written informed consent was obtained and the study was performed in accordance with the Helsinki Declaration. The study eligibility criteria were histologically confirmed rectal adenocarcinoma scheduled to radical surgery alone or CRT followed by surgery, with no prior rectal cancer treatment and age ≥ 18 years. Participants were enrolled between 2013 and 2018, and all images were acquired before treatment start. No patients had mucinous tumours or hip protheses. Patient and tumour characteristics are provided in Table 1.

**Table 1:** Patient and tumour characteristics at time of diagnosis. Except where indicated, data are numbers of patients, with percentages in parentheses.

Variable	Value
No. of patients	23
Gender	
Male	12 (52%)
Female	11 (48%)
Median age, years^a^	66 (50–88)
T stage^b^	
mrT1/2	9 (39%)
mrT3	10 (44%)
mrT4	4 (17%)
N stage^b^	
mrN0	12 (52%)
mrN1	10 (44%)
mrN2	1 (4%)
Median tumour volume [cm^3^]^a,c^	17.7 (4.5–109.9)
Median carcinoembryonic antigen (CEA) [µg/L]^a^	3 (1 – 320)
Preoperative chemoradiotherapy	3 (13%)

a Numbers in parentheses are ranges.

b Assessed by magnetic resonance imaging (MRI) according to the tumour-node-metastasis (TNM) system.

c Assessed by contouring the tumour volume in T2-weighted MR images. The average tumour volumes based on tumour contours by two independent readers are reported.

### MR imaging

Patients were imaged using a 1.5T Philips Achieva MRI scanner (Philips Medical Systems, Best, The Netherlands) with a SENSE Cardiac coil. To reduce bowel peristalsis, a combination of Buscopan (20 mg/mL, 0.5 mL intravenously) and glucagon (1 mg/mL, 1 mL intramuscularly) were administered immediately before the patient was centred in the scanner. Conventional high-resolution fast spin-echo T2-weighted (T2W) images of the pelvic cavity and rectum were obtained in the sagittal and transversal planes as well as perpendicular to the tumour axis, with repetition time (TR) = 2,820 – 3,040 ms, echo time (TE) = 80 ms, acquisition matrix = 256 × 230, reconstructed matrix = 512 × 512, slice thickness = 2.5 mm, number of excitations = 6, echo train length = 20, and in-plane image resolution = 0.35 × 0.35 mm^2^. For research purposes, additional DWIs were acquired using a free breathing, fat-saturated single-shot spin-echo echo-planar imaging sequence with *b*-values = 0, 25, 50, 100, 500, 1,000, and 1,300 s/mm^2^ parallel to the T2W images, with TR = 3,125 ms, TE = 75 ms, acquisition matrix = 80 × 57, reconstructed matrix = 128 × 128, slice thickness = 4 mm, number of excitations = 6, and in-plane image resolution = 1.25 × 1.25 mm^2^. Three orthogonal diffusion gradient directions were used. Phase-encoding was performed in the left–right direction in order to minimise motion artefacts from breathing observed in the anteroposterior direction. All MR images were stored in a dedicated study database. Diagnostic T and N stages were assessed according to international guidelines and the 7th edition TNM staging system [19].

### Quantitative DWI analysis

To evaluate the impact of *b*-value combinations, only the primary tumours were included in the assessment. Guided by DW images, two radiologists with 12 and 5 years of experience independently delineated whole-tumour volumes of interest (VOIs) freehand on the T2W images using the commercially available nICE software package (Nordic NeuroLab, Bergen, Norway). The VOIs were then transferred to the R software environment (R Foundation for Statistical Computing, Vienna, Austria) for analysis of segmentation consistency. If the delineations were found to be consistent based on comparative tests, subsequent analyses were performed using the contours from only one of the radiologists.

Using in-house python scripts, voxel-wise calculations of ADCs within each VOI were performed with different combinations of *b*-values using the monoexponential model: *SI = SI_0_* · *e*^*-b*·*ADC*^, where *SI_0_* represents the signal intensity without diffusion weighting (*b* = 0 s/mm²). As a reference, the biexponential model incorporating all *b*-values was utilised, calculating the tissue diffusion coefficient D and the perfusion-induced coefficient D*: *SI = SI_0_* · *((1 − f)* · e^-b·D^+f · e^-b·D*^). The signal intensity in each voxel within individual VOIs as function of the *b*-value was fitted to the mono- and biexponential models using the Levenberg–Marquardt least square minimisation algorithm, with all *b*-values being equally weighted. Voxels with negative diffusion or perfusion were excluded. The fitting procedure was optimised by implementing an outlier detection rule, using the [Q1 – c*IQD, Q3 + c*IQD] equation, where c was fixed at 1.5, Q1 and Q3 represented the lower and upper quartiles of the data distribution in the VOI, respectively, and IQD = Q3 – Q1 was the interquartile distance. Mean and standard deviation for tumour diffusion and perfusion characteristics were calculated for all VOIs.

### Review of literature

To gain an overview of current practices in *b*-value utilisation, a systematic literature search was conducted in the PubMed database on 19 May 2025. The aim was to identify full-text, English-language articles reporting ADC values in rectal cancer. The search strategy included the terms ‘rectal cancer’, ‘diffusion’, and ‘ADC’, and was designed to retrieve the 10 most recent studies meeting predefined inclusion criteria. Studies were eligible if they reported *b*-values and mean pre-treatment whole-tumour ADC values across the entire rectal cancer patient population, irrespective of treatment outcomes. Exclusion criteria included lack of full-text availability, missing ADC data, non-human studies, evaluation of other cancer types, and studies involving MR-Linac imaging. Titles and abstracts were screened for relevance, followed by full-text review. Studies were assessed in reverse chronological order until 10 eligible publications were identified. From each study, data were extracted regarding the type of exponential model used, the *b*-values applied, and the reported ADC values within segmented tumour volumes. Based on the findings, relevant *b*-value combinations closely aligned with those reported in the literature were incorporated into the analysis.

### Statistical analysis

The intraclass correlation coefficient (ICC) and Bland–Altman plots were used to evaluate interobserver variability in tumour VOI contouring. Shapiro–Wilk tests were applied to test ADC distributions for normality, and subsequently student t-tests were used to assess differences between tumour ADCs calculated from different *b*-value combinations. *P* value less than 0.05 was considered statistically significant. Statistical analysis was performed using *R* software.

## Results

**Figure 1:**
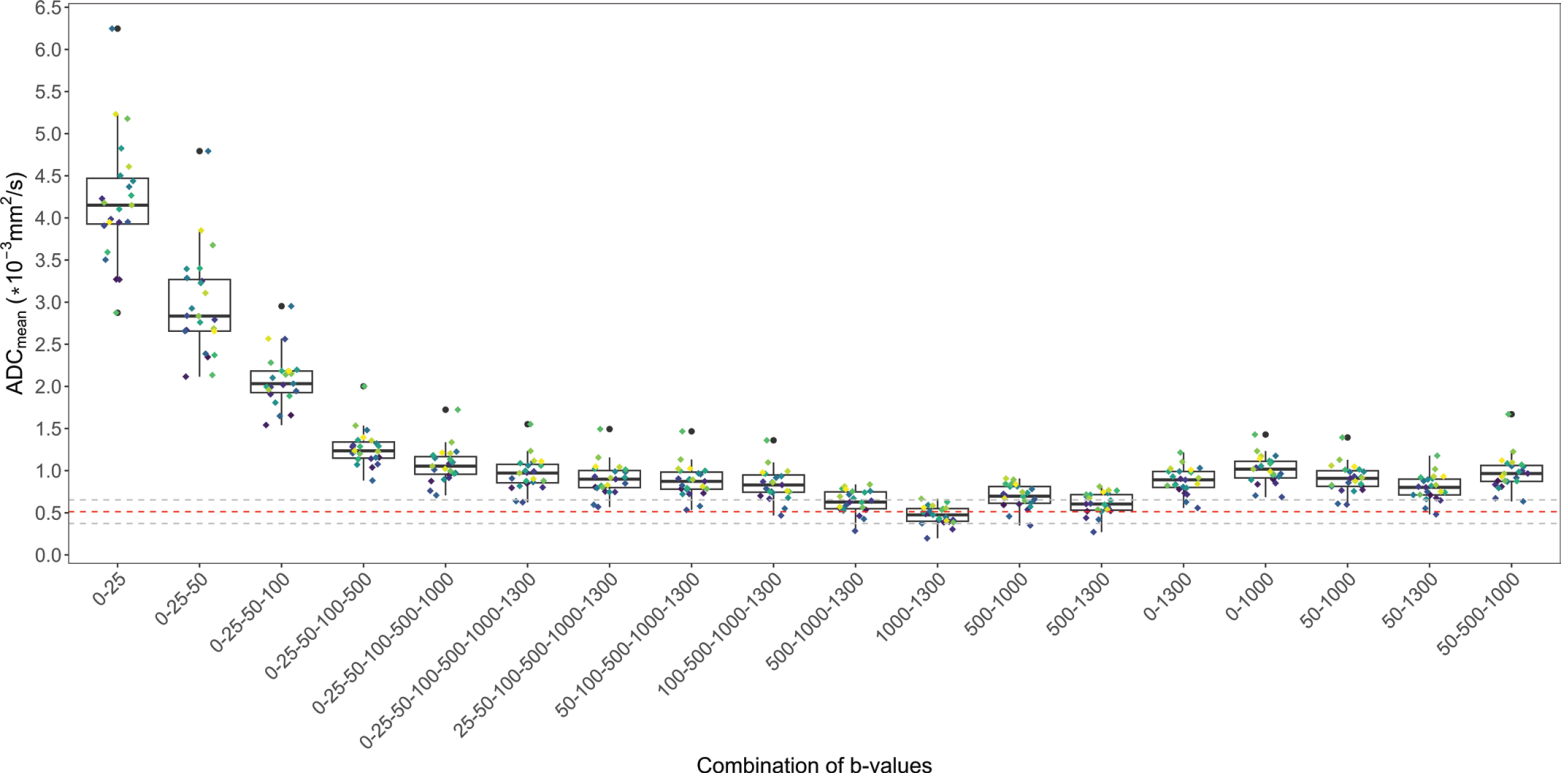
Apparent diffusion coefficient (ADC) vs b-value combination. Individual patients are shown in coloured dots. The dotted lines show mean (red) and standard deviation (grey) of the diffusion value calculated by the biexponential model.

The analysis included 18 *b*-value combinations and uncovered significant variability in tumour ADCs calculated using the monoexponential model, as seen in Table 2. From the biexponential modelling the perfusion-induced diffusion parameter D*, being the signal component mainly arising from contributions at low *b*-values, was found to be about 38-fold higher than the tissue diffusion parameter D. Accordingly, when including the low *b*-values in the monoexponential model the resulting tumour ADCs were significantly overestimated when compared to the reference parameter D from the biexponential modelling. Figure 1 shows a boxplot with coloured dots for individual patients’ tumour ADCs, illustrating that the ADCs varied with the same trend for all patients. The greatest variability was observed in combinations that included only low *b*-values (0–100 s/mm²). In contrast, the use of higher *b*-values, specifically 1,000 and 1,300 s/mm², resulted in both the ADC value closest to the biexponential reference and the smallest variation (Table 2, Figure 1).

**Table 2:** Apparent diffusion coefficients (ADCs) calculated for different b-value combinations. The first 11 combinations are systematic combinations. The 12th and 13th combinations are added to analyse all combinations of values above 100 s/mm2. Combinations 14–18 are close to the recently reported b-value selections.

No	*b*-value combination (s/mm^2^)^a^	Mean ± standard deviation of tumour ADC (× 10^-3^ mm^2^/s)	Deviation from reference ADC^c^ (%)	*p* ^d^
1	0, 25	4.199 ± 0.727	718.1	< 0.001
2	0, 25, 50	2.964 ± 0.611	477.4	< 0.001
3	0, 25, 50,100	2.082 ± 0.314	305.7	< 0.001
4	0, 25, 50, 100, 500	1.267 ± 0.217	146.9	< 0.001
5	0, 25, 50, 100, 500, 1,000	1.069 ± 0.208	108.2	< 0.001
6	0, 25, 50, 100, 500, 1,000, 1,300	0.966 ± 0.198	88.2	< 0.001
7	25, 50, 100, 500, 1,000, 1,300	0.901 ± 0.193	75.6	< 0.001
8	50, 100, 500, 1,000, 1,300	0.878 ± 0.193	71.1	< 0.001
9	100, 500, 1,000, 1,300	0.837 ± 0.187	63.0	< 0.001
10	500, 1,000, 1,300	0.631 ± 0.137	22.9	< 0.001
11	1,000, 1,300	0.470 ± 0.110	-8.5	0.070
12	500, 1,000	0.697 ± 0.140	35.9	< 0.001
13	500, 1,300	0.607 ± 0.131	18.2	0.002
14	0, 1,300	0.884 ± 0.152	72.2	< 0.001
15	0, 1,000	1.011 ± 0.168	97.0	< 0.001
16	50, 1,000	0.906 ± 0.171	76.5	< 0.001
17	50, 1,300	0.797 ± 0.153	55.2	< 0.001
18	50, 500, 1,000	0.973 ± 0.208	89.6	< 0.001
19	0, 25, 50, 100, 500, 1,000, 1,300	0.513 ± 0.140 (D)^b^ 19.371 ± 11.629 (D*)^b^	- 3673.8	- -

a Except for the *b*-value combination at the bottom all calculations were performed using the monoexponential model. For the bottom combination the biexponential model was used.

b The use of the biexponential model separated the contributions from tissue diffusion (D) and perfusion-induced diffusion (D*).

c Deviation calculated as relative difference between mean tumour ADC for each *b*-value combination in the monoexponential model versus mean tumour D from the biexponential model.

d Statistical significance was assessed by students t-test comparing all mean tumour ADCs for each *b*-value combination in the monoexponential model to the mean tumour D from the biexponential model.

**Figure 2:**
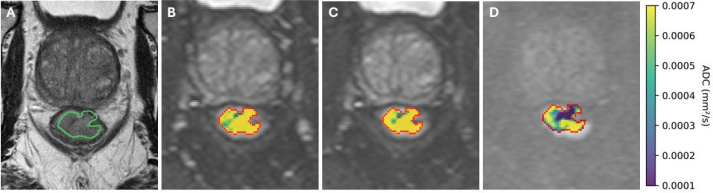
Illustration of apparent diffusion coefficient (ADC) maps using varying b-values. T2-weighted image from an 88-year-old male patient with magnetic resonance-assessed T4 rectal cancer with the tumour region-of-interest contoured in green (A). Corresponding diffusion-weighted image (DWI, b = 0 s/mm^2^) with ADC maps calculated using b = 0, 1,000 s/mm^2^ (B), (DWI, b = 50 s/mm^2^) b = 50, 500, 1,000 s/mm^2^ (C), and (DWI, b = 1,000 s/mm^2^) b = 1,000, 1,300 s/mm^2^ (D).

Figure 2 illustrates the variability in ADC estimation within a tumour slice from a study participant. A T2W image is shown in Figure 2A, alongside tumour ADC maps derived using three different *b*-value combinations (Figure 2B–D). Including low *b*-values in the ADC calculation (Figure 2B–C), the ADC values are noticeably higher compared to when they are excluded (Figure 2D). Given the excellent interobserver agreement, reflected by a mean difference of 0.43 cm³ and an ICC of 0.98 for whole-tumour VOI delineation (see Supplementary Material, Figure S1), contours from a single radiologist (A.N.) were selected for the quantitative diffusion analysis.

A total of 48 articles were retrieved in the literature search. The most recent eligible study was published in April 2025, while the tenth dated back to June 2023, as summarised in Table 3. To enable comparison with the literature, combinations 14–18 in Table 2 were selected to estimate ADCs using *b*-values closely aligned with those recently reported. Notably, all studies incorporated *b*-values below 100 s/mm² in their ADC calculations, despite the known impact of perfusion-related signal decay on the resulting ADC values.

**Table 3:** Heterogeneity in selection of b-values for calculation of apparent diffusion coefficient in rectal cancer. Ten most recent original articles on rectal cancer where *b*-values and mean pretreatment whole-tumour apparent diffusion coefficient (ADC) from the entire patient population (independent of treatment outcome) are reported.

Authors [reference]	*b*-value combinations (s/mm^2^)	Group	Pre-treatment whole-tumour ADC (× 10^-3^ mm^2^/s)
Wang et al. [11]	0, 800	Low grade: High grade:	1.86 (1.49–2.47) 1.34 (1.21–1.72)
Yin et al. [13]	0, 800	LNM negative: LNM positive:	1.57 ± 0.62 1.09 ± 0.29
Wang et al. [12]	0, 800	NLNM-: NLNM+:	1.06 ± 0.22 1.00 ± 0.13
Yang et al. [14]	0, 1,000	Radiologist 1: Radiologist 2:	1.16 ± 0.130 1.15 ± 0.125
Zhou et al. [15]	0, 1,000	KRAS wild type: KRAS mutant:	1.13 ± 0.09 1.08 ± 0.10
Yuan et al. [10]	0, 1,000	pCR: non-pCR:	0.96 ± 0.11 1.06 ± 0.12
Lu et al. [20]	0, 1,000 50, 2,000	*b* = 1000, stage 1: *b* = 1000, stage 2: *b* = 2000, stage 1: *b* = 2000, stage 2:	0.90 ± 0.11 0.83 ± 0.10 0.72 ± 0.08 0.66 ± 0.09
Zhang et al. [21]	0, 1,000 0, ≥1700	ADC_cutoff_: ADC_cutoff_:	1.140^c^ 0.716^c^
Karahacioglu et al. [22]	50, 400, 800	CR: non-CR:	0.77 (0.52–0.98) 0.77 (0.62–1.02)
Zhou et al. [23]	50, 1,000	pCR: non-pCR:	1.09 ± 0.20 1.10 ± 0.21

a ADC given in median (range) or mean ± standard deviation.

b CR: Complete Response, LNM: Lymph Node Metastasis, pCR: pathological Complete Response, KRAS: Kirsten Rat Sarcoma, NLNM: nonenlarged lymph node metastasis.

c No range or standard deviation given.

## Discussion and conclusion

In this study involving multiple *b*-value DWI of patients with rectal cancer, we observed substantial variability in tumour ADC values when calculated using different *b*-value combinations within the monoexponential model. Excluding low *b*-value DW images, susceptible to perfusion-related signal contamination, reduced this variability.

Tumour ADC has emerged as a potentially valuable biomarker for predicting and monitoring response to CRT. Nonetheless, current evidence remains inconsistent, and the technique lacks the precision required to safely guide clinical decisions, such as selecting candidates for organ-preserving treatment approaches. For instance, studies have reported conflicting associations between low pre-treatment tumour ADC values and CRT outcomes. In the studies by Barbaro et al. [24] and Palmisano [25], low pre-treatment ADC predicted poor CRT response, whereas Yuan et al. [10], Lambrecht et al. [26], and Intven et al. [7] found it to be predictive of good response, albeit using different cut-off values and diagnostic accuracies. Notably, the *b*-value combinations used in these studies varied considerably: Barbaro et al. and Yuan et al. employed *b* = 0 and 1,000 s/mm²; Palmisano et al. used *b* = 0, 200, 600, and 1,000 s/mm²; Lambrecht et al. used *b* = 0, 50, 100, 500, 750, and 1,000 s/mm²; and Intven et al. applied *b* = 0, 200, and 800 s/mm². Given the significant influence of *b*-value selection on ADC measurements demonstrated in this study, these choices of *b*-values in the methodology may, at least in part, account for the divergent findings.

Among the combinations tested, those involving two high *b*-values (*b* = 1,000 and 1,300 s/mm²; *b* = 500 and 1,300 s/mm²) resulted in smaller deviations from D compared to the best-performing three-value combination (*b* = 500, 1,000, and 1,300 s/mm²). Notably, this was the only three-value combination that did not include any *b*-values at or below 100 s/mm². Recent advances in MRI technology have made it feasible to incorporate both a greater number and higher range of *b*-values in abdominal imaging, without significantly prolonging scan time or exacerbating motion artefacts. Our findings suggest that excluding low *b*-values (≤ 100 s/mm²) and incorporating high *b*-values (up to at least 1,000 s/mm²) yields ADC values that most closely approximate the true diffusion as determined by biexponential modelling. This is particularly important when the objective is to assess tissue diffusion as a surrogate for tumour cell density. Improved accuracy can be achieved by minimising the influence of perfusion-related effects on the diffusion signal, which are more pronounced at low *b*-values.

When using only high *b*-values (b = 1,000 and b = 1,300 s/mm²), the ADC was underestimated by 8.5%. Due to the exponential signal decay in the monoexponential model, noise can obscure the signal and introduce bias into the ADC estimation as *b*-values increase. Some imaging protocols employ a range of *b*-values to balance sensitivity with the signal-to-noise ratio. However, as illustrated in Figure 1, the inclusion of low *b*-values may still result in an overestimation of the signal.

Comparable studies have been conducted in breast, prostate, and gynaecological cancers, and overall, their findings are consistent with ours [27–29]. Supporting this, a consensus and recommendation paper on the role of DWI as a cancer biomarker also advised that only *b*-values of ≥ 100 s/mm² should be used for quantitative DWI [20]. Despite this, many clinical studies continue to incorporate low *b*-values in ADC calculations. To fully realise the potential of tumour ADC in predicting and evaluating response to CRT – particularly through the identification of robust cut-off values with high diagnostic accuracy – the standardisation of *b*-value selection, image processing protocols, and ADC calculation methods is critically needed. Only through such standardisation can the true clinical utility of tumour ADC in the individualisation of rectal cancer treatment be established, in accordance with recommended investigational pathways for promising radiological biomarkers, including systematic evaluation in clinical outcome trials [30].

The study is limited by the investigation of only a representative selection of *b*-value combinations, rather than exploring the full range of possibilities. Acquiring additional *b*-values above 100 could have further supported the identification of an optimal *b*-value combination for quantitative ADC analysis. Moreover, although 18 different combinations were assessed, the analysis was conducted on a subset of 23 patients from the OxyTarget cohort. This group was considered adequate to reflect ADC variability, while broad inclusion criteria, prospective enrolment, and fixed MRI scheduling ensured a diverse and representative sample. Furthermore, the study was limited to a single scanner model. Although the optimal combination of *b*-values may vary between vendors, the pronounced influence of perfusion effects introduced by low *b*-values – supported by findings from comparable studies [27–29] – suggests that the main conclusions are likely to be generalisable across different scanner types.

In conclusion, the ADC of rectal tumours, as calculated using the monoexponential model, is highly dependent on the selection of *b*-values. Employing higher *b*-values that minimise the influence of blood perfusion (i.e. excluding low *b*-values ≤ 100 s/mm²) significantly reduces uncertainty in ADC estimation. Under these conditions, the ADC more accurately reflects the true tissue diffusion, D, as derived from the biexponential model. Our findings support the use of *b*-values exceeding 100 s/mm², ideally in combination with a high *b*-value of at least 1,000 s/mm², when assessing diffusion using the monoexponential model. For reliable quantitative comparisons of ADC values, it is strongly recommended to employ consistent *b*-value combinations across studies.

## Supplementary Material



## Data Availability

The data are available upon reasonable request to the authors.
