# Supplementary material for: Low b-values in apparent diffusion coefficient calculations overestimate diffusion in rectal cancer
Source: Acta Oncol. 2025 Oct 19;64:44028. doi: 10.2340/1651-226X.2025.44028 (PMC12553313; doi:10.2340/1651-226X.2025.44028)
Supplement: Supplementary file 1 [file AO-64-44028-s1.pdf]

Supplementary material has been published as submitted. It has not been copyedited, or typeset by Acta Oncologica

Supplementary material

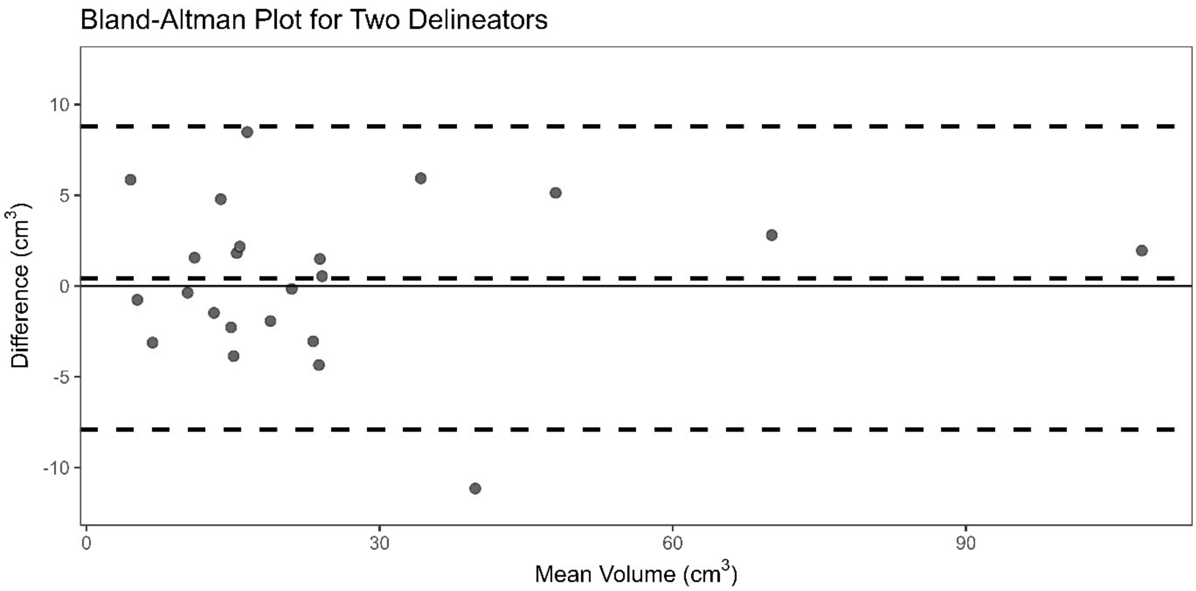

Figure 3: Bland-Altman plot for region of interest delineation. Includes delineations from two experienced radiologists on 23 patients, contoured using both T2W and DW images as guidance. Dotted lines represent mean difference (0.43 cm^3) ±1.96 standard deviation (8.79 cm^3, -7.92 cm^3).
